# Strategies for obtaining unpublished drug trial data: a qualitative interview study

**DOI:** 10.1186/2046-4053-2-31

**Published:** 2013-05-16

**Authors:** Nicole Wolfe, Peter C Gøtzsche, Lisa Bero

**Affiliations:** 1 Department of Social and Behavioral Sciences, University of California, Box 0612, 3333 California St, Suite 455, San Francisco, CA 94118, USA; 2 Nordic Cochrane Centre, Rigshospitalet, Dept 7811, Blegdamsvej 9, DK-2100 Copenhagen, Denmark; 3 Department of Clinical Pharmacy Institute for Health Policy Studies, University of California, Box 0613, 3333 California St, Suite420, San Francisco, CA 94118, USA

**Keywords:** Systematic review, Meta-analysis, The Cochrane Collaboration, Reporting bias, Publication bias

## Abstract

**Background:**

Authors of systematic reviews have difficulty obtaining unpublished data for their reviews. This project aimed to provide an in-depth description of the experiences of authors in searching for and gaining access to unpublished data for their systematic reviews, and to give guidance on best practices for identifying, obtaining and using unpublished data.

**Methods:**

This is a qualitative study analyzing in-depth interviews with authors of systematic reviews who have published Cochrane reviews or published systematic reviews outside of The Cochrane Library. We included participants who 1) were the first or senior author of a published systematic review of a drug intervention, 2) had expertise in conducting systematic reviews, searching for data, and assessing methodological biases, and 3) were able to participate in an interview in English. We used non-random sampling techniques to identify potential participants. Eighteen Cochrane authors were contacted and 16 agreed to be interviewed (89% response rate). Twenty-four non-Cochrane authors were contacted and 16 were interviewed (67% response rate).

**Results:**

Respondents had different understandings of what was meant by unpublished data, including specific outcomes and methodological details. Contacting study authors was the most common method used to obtain unpublished data and the value of regulatory agencies as a data source was underappreciated. Using the data obtained was time consuming and labor intensive. Respondents described the collaboration with other colleagues and/or students required to organize, manage and use the data in their reviews, generally developing and using templates, spreadsheets and computer programs for data extraction and analysis. Respondents had a shared belief that data should be accessible but some had concerns about sharing their own data. Respondents believed that obtaining unpublished data for reviews has important public health implications. There was widespread support for government intervention to ensure open access to trial data.

**Conclusions:**

Respondents uniformly agreed that the benefit of identifying unpublished data was worth the effort and was necessary to identify the true harms and benefits of drugs. Recent actions by government, such as increased availability of trial data from the European Medicines Agency, may make it easier to acquire critical drug trial data.

## Background

Selective reporting of data from clinical trials, particularly drug trials, has been well documented [1-3]. Some trials are not published at all while others are incompletely published, including only those results that are most positive for the drug being studied. Studies of selective reporting bias have generally compared the results of unpublished comprehensive study reports or trial protocols with published papers. These investigations have used a variety of sources to identify unpublished studies and outcomes. Researchers have found that the full reports of drug studies identified by searching regulatory agency databases have often not been published, or contain far more data than published papers [4-6]. Legal actions with drug companies have identified many unpublished trials or data [7-9]. Investigators have also identified unpublished trials and data by searching for trial protocols [10,11].

The incomplete or total lack of reporting of drug trials is so common that our perceptions of the true benefits and harms of drugs are generally much too positive [5,6,9,12,13]. There have been prominent cases of well known drugs, such as gabapentin, oseltamivir, and rofecoxib where the analysis of unpublished data revealed important insights about the benefits and harms of those drugs not previously identified in their initial publications [14].Therefore, it is critical that systematic reviews of drugs, which are often used as the basis for clinical practice guidelines, identify and include unpublished data from drug trials. The Cochrane Collaboration is a major producer of rigorous systematic reviews of health care interventions, but only 12% of Cochrane reviews from 2000 to 2006 included unpublished trials [15]. *The Cochrane Handbook for Systematic Reviews of Interventions* suggests identifying unpublished data by contacting experts, pharmaceutical companies, and national and international trial registers [16]. No specific guidance for searching for drug trials is provided and specific sources of drug trial data such as regulatory agencies or drug company archives resulting from legal settlements are not mentioned. In addition, little advice is provided in *The Cochrane Handbook* or elsewhere about strategies for obtaining the data from different sources.

The objective of this project is to provide an in-depth description of the experiences of researchers doing systematic reviews in searching for and gaining access to unpublished data. Additionally, this project aims to give guidance on best practices for identifying, obtaining, and using unpublished data from a variety of sources.

## Methods

### Design

This is a qualitative study analyzing in-depth interviews with authors of systematic reviews of drugs.

### Sampling

We interviewed authors of systematic reviews who have published Cochrane reviews or published systematic reviews outside of The Cochrane Library. We included participants who 1) were the first or senior author of a completed, published systematic review of a drug intervention, 2) had expertise in conducting systematic reviews and meta-analyses, searching for published and unpublished data, and assessing methodological biases, and 3) were able to participate in an interview in English. The expertise of the participant was confirmed during the initial contact. We excluded potential participants who were authors of reviews of devices or interventions other than drugs.

**Figure 1:**
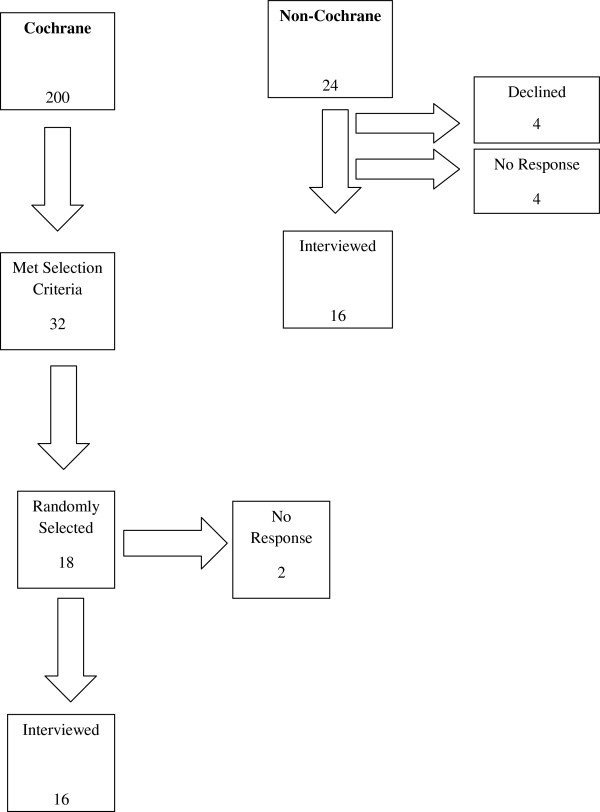
Participant flowchart.

We used non-random sampling techniques to identify potential participants who had the knowledge and professional experience to address the aims of our study. We selected systematic review authors who met our inclusion criteria with the aim of achieving diversity of research topic area and geographical location. Our sample size was determined by guidelines from the qualitative methodology literature that suggests an adequate sample size for qualitative research should be 20 to 40 people [17-19]. In a prior cross-sectional, on-line survey [20], 200 Cochrane authors agreed to be interviewed for this study and 32 met our inclusion criteria. We randomly selected 18 of these for initial contact; 2 did not respond after two attempts and 16 agreed to be interviewed (89% response rate). Fifteen were first authors on one or more Cochrane reviews, while the sixteenth was a co-author of several Cochrane reviews (See Figure 1).

The researchers outside of the Cochrane Collaboration were chosen using non-random and snowball sampling. Snowball sampling relies on participants in the study or those with knowledge of the study, to identify other people they believe could speak to the questions raised in the study. The investigators for this project identified authors of published systematic reviews who met our inclusion criteria and one researcher who was contacted for the study referred us to two of his colleagues. In an effort to geographically diversify this sample, we also sent an e-mail to colleagues asking for researchers outside of the United States and Europe who would fit our selection criteria.

Twenty-four non-Cochrane authors were contacted; four replied that they could not participate, four did not respond, and 16 were interviewed (67% response rate). Of the four who could not participate, two were going to be out of town, one was too busy with professional commitments, and the fourth did not feel he was qualified to speak to the aims of our study.

### Interview procedures

All potential interviewees were contacted up to two times via e-mail. The introductory e-mail explained the purpose of the study, why the interviewee was being contacted, potential risks, and procedures for assuring anonymity. At the beginning of each interview, the participant was asked to provide verbal consent and permission for the interview to be recorded. Our study was approved by the University of California, San Francisco Committee on Human Research (# 11–08289).

Of the 32 interviews conducted, one was conducted in person, while the others were conducted via Skype (5 face-to-face video, 26 audio only). All interviews were digitally recorded and uploaded to a secure server accessible by the authors.

Nineteen interviews were transcribed. The cost of transcription prevented us from transcribing all interviews, so only those that provided unique information were transcribed. Extensive notes were taken from the interviews that were not transcribed, and those notes were coded in the same way as the transcribed interviews.

The interviews were conducted between June and September 2012.

### Interview content

We used a semi-structured interview format to enable researchers to describe their experiences in their own words [17,21]. We used an interview guide to ask specific, yet open-ended questions, which could then be followed with more probing questions. See Additional file 1 for interview guide. The interview questions fell into four main categories:

1. Professional background/history of systematic reviews or work in this area

2. Understanding what is meant by unpublished data

3. General methods for obtaining and using unpublished data

4. Obtaining and using unpublished data, with a specific review as an example.

### Analytical method

The transcribed interviews and notes were initially coded using an ‘open coding’ scheme that developed iteratively from the text. This process helped to identify the major themes that emerge from the data [17]. The codes were then assigned to pieces of text to organize the data. Once the open coding process was complete and the final set of codes was developed, we systematically coded the content of all interviews using the iteratively developed coding scheme. This allowed us to connect the themes to understand better the interrelationships among them [17,22]. The interviews were imported into the qualitative data management software program, Atlas.ti, to organize and manage the qualitative data. We report common themes, as well as areas where there was variability in responses.

## Results

### Participant characteristics

As shown in Table 1, the participants were from six geographical regions in eleven countries. Greater geographical diversity was achieved among the Cochrane author sample than the non-Cochrane sample. Of the 32 respondents, 66% (21) had M.D. degrees, six of those had a joint medical and graduate research degree; 44% (14) were women.

**Table 1:** Participant characteristics (n = 32)

**Variable**	**Number (%)**
Gender	
Female	14 (44)
Geographical region	
North America	13 (41)
South America	1 (3)
Europe	12 (38)
Australasia	4 (13)
Middle East	1 (3)
Africa	1 (3)
US	13(41)
UK	4(12.5 )
Switzerland	4 (12.5)
Italy	2 (6)
Denmark	1 (3)
France	1 (3)
South Africa	1 (3)
Israel	1(3)
Japan	1(3)
Venezuela	1(3)
Australia	3(10)
Degree^a^	
MD	21 (66)
Graduate research degree^b^	16 (50)

^a^Numbers may add to more than 32 because some respondents had multiple degrees;^b^includes PhD, DrPhil, DrMedSci.

### Major themes

Our study uncovered several major themes. First, study respondents had different understandings of what was meant by unpublished data. Second, contacting study authors was the most common method used to obtain unpublished data and the value of regulatory agencies as a data source was underappreciated. Third, using the data obtained was time consuming and labor intensive, and respondents described a variety of methods to organize, manage, and use the data in their reviews. Fourth, respondents had a shared belief that data should be accessible, but some had concerns about sharing their own data. Fifth, respondents believed that obtaining unpublished data for reviews has important public health implications. Lastly, there was widespread support for government intervention to ensure open access to trial data.

### Definition of unpublished data

Most respondents distinguished between entire unpublished studies and data that were missing from published studies and considered both to be unpublished data. One respondent discussed this saying, ‘the main problem is that we can’t get access to unpublished studies. We don’t even know that they exist and for studies we know about, data are often missing.’ Another respondent noted ‘they’re [trials] never published, they’re never made available, they’re not out there to inform practice.’

Some respondents felt that unpublished data are any data not included in a peer reviewed journal article. Others believed that unpublished meant any data that were not publically available. Therefore, abstracts, conference proceedings, and other ‘grey literature’ were considered unpublished. Others disagreed and believed that abstracts were published data. For these participants, unpublished meant data that could not be publicly obtained in print. One respondent discussed why it is important to look outside of peer-reviewed journals, ‘we also want to be aware of what trials are out there and may be presented at meetings because, you know, journals may have long publication times, but if you’ve got a meeting abstract and you can work a little bit with the author, often you can include some of that data.’

Even when relevant studies were identified, respondents reported that they were missing key pieces of data to complete their reviews. In this instance, unpublished data were described as the specific outcomes that were not included in any publicly available published article of the drug trial.

Standard deviations were mentioned frequently as necessary pieces of data that were often missing from published reports. One respondent described unpublished data as being more than outcome data, and included ‘details of the methodology, data that would go in a Cochrane risk of bias table, and any results not mentioned in the report.’

### Sources of unpublished data

The respondents’ choices of data sources are shown in Table 2. Our analysis of the interview data suggests that these choices were influenced by how they defined unpublished data and whether they believed a source had the data and would be willing to share them. The most frequently mentioned source of unpublished data was study authors. All respondents contacted study authors, yet many did not go further because they did not know that the data might be available elsewhere or they did not believe they would be able to gain access to the data.

**Table 2:** Advantages and disadvantages of sources of unpublished data described by interview respondents

**Sources of unpublished data**	**Advantages**	**Disadvantages**
Study authors	They have the data	• Not always responsive
		• Want to keep the data to themselves for their own publications
Drug companies	• Have complete clinical study reports	• Rarely responsive
		• Want to know what you want to do with the data
Regulatory agencies	• Includes summary data from clinical trials	• Not a user friendly interface
		• People do not know what information/data are available on the agencies websites
		• Not as much information about older drugs
		• Data on phase 4 studies often missing
Grey literature	Provide information about studies that may be published, for example, presented at conferences.	• Does not provide key pieces of outcome data
		• Rarely peer reviewed
Law firms - class action suits in the US	• Can uncover all of the data for a given drug	• Happens for few drugs
		• Tens of thousands of pages of documents to search
		• Judges must agree to release the data
Grant organizations	• Provide a list of all studies they sponsor	• Sponsor a small number of studies
Marketing materials from companies and financial reviews	• More or different data may appear in financial publications compared to the scientific literature	• Can suggest that unpublished data exist, but the data must still be obtained
The media – journalists, press officers	• Companies will respond to media pressure	• Data may not be complete
Clinical trial registries	• Provide awareness of all of the trials being conducted on a certain drug in a certain area	• Results data are generally not available
		• Some registries do not contain registration of Phase 1 or 4 trials

Some respondents turned to trial registries to help identify entire unpublished trials. The benefit of the trial registry is that it can help a researcher identify trials for which no outcomes have been reported. The shortcomings are that they do not provide any data and there is no contact person listed.

Others sources that were less frequently mentioned were grey literature, which, depending on the respondent, might include non peer-reviewed publications, abstracts, conference proceedings, or dissertations. One respondent mentioned law firms involved in class action lawsuits as a data source, but noted that while a class action lawsuit can reveal all of the trial data, they do not occur very often and cannot be considered a source for many drugs. The media, press releases, and grant organizations were also mentioned as potential sources for unpublished data.

### Strategies for obtaining the data

#### Contacting the study author

Respondents used similar processes to approach study authors about obtaining unpublished data and believed two methods were most successful. The first was keeping the initial e-mail to the study author descriptive, friendly, and concise. Making it easy on the study authors was important in receiving the data because, as one respondent stated, ‘it’s just a matter of understanding that people, even if they want to collaborate, they don’t have a lot of time to do that. So, if you make their life more complicated, they’ll say, “Okay, why do I need this problem?”’ The second method that increased response rates was to try to establish a personal connection with the author. Additionally, respondents noted that more senior authors were more likely to be successful in obtaining data. Respondents believed they would also have more success if their request came from a larger organization with which the researcher was affiliated (for example, a Cochrane Center or the World Health Organization) rather than contacting the author as ‘a lone wolf.’ Generally, respondents would only contact a study author twice if they received no response. However, others believed that persistence was important. One respondent contacted unresponsive study authors ‘every six months for two to three years, until the review was completed.’

#### Unresponsiveness or refusal to share data

There were three main reasons provided to our respondents as to why the study author(s) they contacted would not or did not provide the data our respondents were seeking. The first was that the study author had moved from one institution to another and no longer had access to the data. The second reason was that the study author did not have the resources to search for or gather the data. Two respondents discussed the need to obtain extra funding to get unpublished data. One paid study authors directly for the data, which he stated increased the response rate considerably.

The third reason noted by our respondents was that authors wanted to maintain control over the data for their own publications and professional advancement. One respondent described it this way, ‘clinical trialists are quite willing to share their data if they had published their own results.’ Although respondents did not agree with this practice, as academicians, they shared similar concerns. One respondent stated, ‘I could understand if you put a lot of energy and resources into gathering data that you don’t want to just turn it over to someone else to publish all of your papers with.’

#### Pharmaceutical companies

Researchers who were unable to obtain the data from the study author believed that the potentially most fruitful next step would be to contact the pharmaceutical company that sponsored the trial. However, due to past negative experiences or perception, many respondents did not believe they would be granted access to the data and concluded that the attempt would not be worth the effort. While Cochrane authors were less likely to contact drug companies, there was a general distrust of the pharmaceutical industry by all respondents. One respondent stated, ‘an overture to a pharmaceutical company to get data from them is not likely to meet with any kind of success. You would have to – I would assume that anything I got back from them was not likely to be valid or that you couldn’t actually depend on it.’ Drug companies often responded with reasons as to why the data could not be released. One respondent stated that she was told by multiple company representatives that the ‘quality of the studies is just too poor to do meaningful analysis and based on that argument we did not get the data.’ Even when companies were required through legal action to make data available, most respondents believed it would be difficult to make the analysis free of industry influence since some settlements required that company employees still be involved as consultants.

One of the most informative and comprehensive documents that a reviewer can obtain from the pharmaceutical company is the clinical study report. The ‘clinical study reports contain hundreds to several thousand pages and at an aggregate level contain everything.’ However, the immense size of the reports makes the process of finding the needed data very laborious and time consuming. The size also allows unflattering data to be hidden among the thousands of pages. One respondent discussed how he had ‘no doubt’ that the large size of the clinical study report was a ‘deliberate tactic that drug companies use that they drown us with data. They submit so many thousands of pages on their clinical trials that no one in the whole world will ever read all this. And there are examples that they have hidden, even deaths, deeply inside a report. And there is not any big chance that anybody will ever find it.’

Despite the size of clinical study reports, one respondent described them as ‘formulaic’ and said that if one understands the formula, then one can easily navigate through the documents. For example, a respondent stated, ‘once you’ve got your head around the structure of one clinical trial report from a company, and a drug, and topic then by in large you know your way around most of the others…sometime it’ll be as simple as table twenty one is always telling you about death… almost all of them have got extensive indices at the front and/or back… almost all of them as well have very extensive sections dealing with the methodology, how missing data were dealt with and so on. And when it comes to significant and severe adverse events they’ll usually have individual patient narratives tucked away in the appendices and one can look and search for those as well.’

Another respondent conceded that these reports are helpful, and ‘your means of verification with the clinical study reports are far higher’ than in a peer-reviewed journal article. He did however, feel that ‘the clinical study report is a commercial document…it sells the drug to the regulators, so you shouldn’t be trusting it.’

Some respondents believed that if you didn’t work with companies you would be cut off from obtaining data. Respondents who had success with drug companies had usually been involved in a review supported by a drug company that agreed to make data available. But even those who had been involved in a review with a cooperating drug company may not have success obtaining data for another review.

While most of our respondents worked in academia, one who currently works for a pharmaceutical company had a different perspective: ‘the pharmaceutical companies in general do allow access to the data, but not for anyone. So, typically what would happen is there would be a protocol, it would be reviewed by an internal committee, and there would be an assessment of the scientific rigor of the work, and the capability of the investigator. To provide someone with data requires a fair bit of work. And, so there would be an assessment of is there a good trade off for the amount of work required to provide the data and the expected utility of the data once completed.’

#### Regulatory agencies

Respondents were generally unaware of data from regulatory agencies. The Food and Drug Administration (FDA) and the European Medicines Agency (EMA) were the two regulatory agencies mentioned by respondents. Reviewers saw the FDA as a particularly valuable resource because the FDA has data and analyses that do not get published in peer-reviewed journals. One respondent outside of the United States had high praise for the FDA, stating, ‘the FDA is brilliant in the last few years because of what it does and the way it treats data… What happens now is that the FDA will perform its own analyses on the company’s data, they won’t just rely anymore on the company’s analyses of data and that analysis includes an analysis of the individual patient level and it includes an analysis, which we think is more appropriate where they use a variety of different techniques for dealing with data when patients drop out of studies.’

Since the regulatory agency has the documentation necessary for approval, one respondent believed that ‘having access to regulatory agency submissions is really the kind of gold standard of information on results because it’s the full report of all the analyses that have been done and it’s the full protocol that’s available through those submissions.’ However, respondents noted a number of major limitations of the data available from the FDA website: ‘a lot of it is redacted for proprietary reasons, and it’s not necessarily posted in the most timely fashion or in the most convenient format.’ In addition, the FDA website does not include raw data, complete data on harms, or all post-approval studies. Individual case report forms are not readily available, although summaries of these case reports are provided by the FDA.

The FDA’s site is not user friendly. One respondent found ‘it very hard when I go to the FDA site to find reports. I really have to seemingly dig around a long time, and I suspect a lot of people don’t even bother.’ One respondent felt that the site was so difficult to navigate, and the format of the data so unwieldy, that she preferred not to use it as a source of data. One reviewer who acknowledged that searching the FDA site is ‘a daunting task’ noted that experience made it easier and that reviewers could be trained to use the site efficiently. One respondent was optimistic about the direction the regulatory agencies were heading with regard to making data more available. He said, ‘they have become more accepting of the fact that it’s really illogical and unfair that they should have access to all the information and then to approve the drug or device, and then those of us who actually use or prescribe those interventions then only have access to a biased subset of that information.’

### Using the data in reviews

Respondents discussed the processes they used to organize and manage the data that they obtained. One respondent discussed the collaboration required after receiving data from a drug company that were a ‘complete mess. I had no idea what was going on. Fortunately, it being [Academic institution], a few very clever people around, one of whom was an Associate who was an absolute whiz kid at spread sheets and artificial intelligence and he had mechanisms which allowed us to make sense of the data.’ Another respondent described the process of cleaning the data for use in the review as ‘a huge project. I have had two full-time researchers working on this for a full year now. So we are trying to develop the methods whereby we can digest these thousands of pages without as I told you having to read every word in them.’

The process for extracting usable data can span months or years. One respondent described the process with his research team whereby three of them ‘sat in my room with, I think, three or four computers, all big screens running simultaneously, looking through the data - checking and double checking, triple checking each other and entering data into our Pro forma sheets at the same time. So we just got it done while we were all concentrating on it.’ Another respondent, working under a deadline, had received all of the data, and then checked ‘data for 53 studies, 14,000 patients, then every study was checked by a statistician, I mean the complete IPD (individual patient data) data…we were working night and day, night and day because that was a special project.’

Sometimes the amount of data obtained was simply too much. One respondent was told he could receive 70 meters of paper from a regulatory agency and ‘gave up.’ Another respondent who had to manually go through paperwork felt that it was ultimately ‘not worth doing that. It was a good three days work for a table of data.’ The respondents felt that it was important to be discerning about time spent gathering certain data and weigh that against how critical those data are for that review.

### Beliefs about unpublished data

The respondents expressed a variety of views on collaboration and willingness to share data with other researchers, language barriers, the role of systematic reviews and their impact on public health, and ideas about how the accessibility of data can be increased.

#### Collaboration

Most of the respondents would share data with other researchers when asked and believed that data should be available. However, some respondents who were also trialists were unwilling to share their own data or collaborate with other trialists. One respondent thought the main barrier to data sharing was a lack of collaboration among researchers, and he found it ‘singularly unhelpful to work with people outside [of his institution].’ Others felt ‘uncomfortable’ about sharing data because data could be misrepresented or inappropriately analyzed *post hoc*. While some believed that data should be available, they felt that the availability should be limited to qualified researchers who could demonstrate how the data would be used. Others felt that unpublished data should be widely available to the public with one suggesting that The Cochrane Collaboration establish a database of all unpublished data collected for Cochrane Reviews.

#### Language issues

Language was viewed as a barrier. One respondent said that ‘for better or for worse, English probably is the common currency in terms of scientific publication, and, so most of the best scientists want their work published in English.’ Therefore, non-English language speakers are at a disadvantage when it comes to obtaining data from English-language publications.

There were differing opinions as to how the inclusion of non-English publications would affect the outcome of reviews. Some respondents did not think it was worth the effort to obtain data published in languages other than English, while others felt that it was important not to exclude those studies.

#### Public health

All of the respondents believed that their efforts to obtain unpublished data were important. Many respondents agreed that drug trial data should be more easily accessible to the public. One respondent stated that, ‘we should have the totality of the information available to us and if there are studies which have been done and they are reasonable studies, which could help make decisions either about the effectiveness or harms of medicines then they damn well ought to be in the public domain ‘cause it’s the public that are taking the tablets.’ Another respondent also believed these data should be public, and said ‘This idea that they contain material that the company should be able to keep private is absurd. Human lives were involved in these trials and I think that information should be public.’Another respondent noted that ‘patients would be shocked’ to know that data were unavailable to researchers.

Others stated that without access to unpublished data, the true harms and benefits of many drugs would never be known. Respondents noted that conclusions of drug reviews that do not include unpublished data are ‘dangerous,’ with one noting, ‘peer review journal articles represent a cherry-picked subset of what went on. And I think clinicians and researchers need to know the whole story and not just this sanitized cherry-picked version of the truth.’ One respondent felt that The Cochrane Collaboration was impeding progress by publishing reviews that do not have the full data on a drug, noting ‘we don’t expect Cochrane reviews to be biased towards a certain product, but they are.’ He suggested that Cochrane reviews should ‘come to the conclusion that they can’t get the data’ rather than a conclusion about the efficacy of a drug.

Some of the respondents described how their reviews changed prescribing practices after they exposed harms by including unpublished data. One respondent’s review caused the drug company to remove the drug from the market and cease all ongoing clinical trials. Some respondents felt that difficulty in obtaining data caused unacceptable delays of public health significance. One researcher who was finally able to obtain data from the FDA after three years of repeated Freedom of Information (FOIA) requests stated simply, ‘I think my review is ten years too late.’ The results of his review demonstrated harm at the high dose of the drug. Another respondent who has been a vocal critic of industry felt that his work had negatively impacted public health and believed, ‘I’ve probably increased the sales of drugs. Companies can use critics to increase sales of their drugs. I don’t think I’ve made any difference or whatever, maybe made things worse’.

#### Ways to increase data access

All of the respondents believed that trial data should be made available and that access should be increased but there were differing opinions as to the best way to make this happen. There was widespread support for government intervention and regulation. One respondent described how government intervention could help, ‘it’s very easy if you want to, to demand as a condition for having approval of doing the trial, you need to provide all the data within a certain amount of time… And if you don’t provide the totality of the data, then there could be sanctions like if it is a drug company, the sanction could be that they are not allowed to do anymore trials.’ Another respondent concurred that it ‘would be a great thing for the government to manage and make accessible.’ One US respondent felt that political will could facilitate access to data and found it necessary to ‘get a Senator or Congressman to get it for you.’

There were differing opinions, though, as to who could access the data. Some felt that it should be publicly available to anyone without any barriers. Others felt that the process should be more discerning, with data available to scientists and researchers who could understand and make sense of the data as well as demonstrate how they would use the data, which could involve submitting a protocol for the review.

## Discussion

Researchers with experience searching for unpublished drug trial data uniformly agreed that finding unpublished data has important public health consequences in terms of identifying the true harms and benefits of drugs and improving the reliability of systematic reviews [5,6,12,13]. They also recognized that obtaining the data takes time and effort that could delay the public health impact. The respondents felt that reviews that were dependent on data from drug company sponsored trials and did not contain unpublished data could not reach accurate conclusions but at the same time they distrusted drug companies as a source of data. Our respondents identified several challenges to obtaining unpublished data, including nonresponsive study authors, language difficulties, and unawareness of data from drug regulatory authorities. After unpublished data were indentified, our respondents found that considerable effort was needed to get the data into a format that could be used in the reviews. Not only does the numerical data need to be available, but reviewers must have sufficient information about the characteristics of the studies with unpublished data in order to be able to assess the risks of bias of these studies.

Our study has limitations associated with qualitative interview studies. Although our purposive sample selected researchers who had experience with searching for unpublished data who worked in a variety of topic and geographical areas, our findings may not be generalizable to all authors of systematic reviews. However, the suggestions given by our respondents can be used and tested by systematic review authors without a negative impact to their review. In fact, the lessons learned through this project can serve to strengthen and improve the process for conducting systematic reviews. Our high response rate and openness of the interviewees suggests a willingness to address the topic of searching for unpublished drug trial data and a desire to change the current paradigm.

Researchers, particularly Cochrane authors, have traditionally contacted study authors to obtain missing data. However, the difficulties experienced by our interviewees suggest a need for greater accountability. Trial data that are maintained in accessible databases or study reports should be available long after authors are out of contact via institutional mechanisms for data sharing [23].

A paradoxical finding of our study is that while all respondents demanded access to unpublished data for their reviews, some expressed unwillingness to share their own trial data. Researchers are concerned that their own data will be misinterpreted or analyzed inappropriately. However, if the data are made freely available and the analyses are transparent, it will be possible to debate any conflicting findings in an open scientific discourse. Thus, not only should data be accessible but their analysis should also be transparent. Given the competitive climate in research, publication, and fundraising, researchers who voluntarily share their data when such sharing is not required for everyone may be at a disadvantage. Therefore, standards for data sharing should be uniform and universally applied.

Respondents who had obtained data from regulatory agencies found the experience to be well worth the effort. However, data from a recent quantitative survey suggests that drug regulatory authorities are underutilized as a source of drug trial data [20]. Access to data from regulatory authorities could be improved by standardizing the reporting of data, including more data on harms, and including individual patient data. While most of our respondents had experience with obtaining data from the US FDA, the EMA may become a more valuable data source in the future. In 2010, the Nordic Cochrane Centre obtained a break-through at the EMA after having complained to the European ombudsman that the agency refused to provide protocols and clinical study reports to the Centre [24]. This created an important precedent, as everyone can now obtain such access in accordance with the principles on which the EU is founded. Other drug agencies should follow suit, as the traditional secrecy is not in the patients’ interest and cannot be defended ethically [25].

Difficulties were often encountered when attempts were made to obtain data from drug companies. Respondents with prior experience working with drug companies were more likely to obtain data but even they stressed that users of the data must be aware that industry controls the data, and this is another reason why raw data for independent analyses should be available. Difficulty in obtaining data from drug companies could be avoided by enforcing legal requirements to provide the data.

The *Cochrane Handbook for Systematic Reviews of Interventions*[16] suggests identifying unpublished data by contacting experts, pharmaceutical companies, national and international trial registers (for example, clinicaltrials.gov), and other specific trial registers. No specific guidance for searching for drug trials is provided and sources of drug trial data, such as legal settlements, regulatory agencies, human subject approvals, and annual reports of funding agencies are not mentioned. In addition, little advice is provided in The *Cochrane Handbook* or elsewhere about strategies for obtaining the data from different data sources. Future revisions of the *Cochrane Handbook* should take into account reviewers’ experience with obtaining unpublished drug study data from regulatory agencies.

## Conclusion

Our findings suggest that the burden of responsibility for making clinical drug trial data available to researchers should not lie with individual study authors or with sponsoring companies, but with government authorities. Some respondents suggested advocacy approaches such as doctors refusing to prescribe drugs or patients refusing to enroll in clinical trials until all the data become available. However, drug trial research is an international activity and these advocacy efforts would be difficult to implement globally. Others felt that change needed to be made at the highest levels, whereby large entities such as the World Health Organization, national health departments, state agencies, and even large managed care organizations could require full availability of data for inclusion of drugs in their formularies. Government intervention could take the form of enforcing requirements to make all data available in clinical trial registries, granting ethics approval for trials only when data from previous trials have been published, and making all data from drug regulatory authorities available. Some of this is not far away. At a workshop at the EMA on 22 November 2012, EMA announced that it will in future ask the companies to submit not only the study protocols and the clinical study reports but also the raw anonymized patient data in a statistically useful format, which it will make available to the public [26].

## Abbreviations

EMA: European Medicines Agency; FDA: Food and Drug Administration.

## Competing interests

The authors declare that they have no competing interests.

## Authors’ contributions

NW collected all qualitative data, conducted data analysis, and drafted the manuscript. PG participated in the conception and design of the study and assisted with manuscript revision. LB participated in the conception and design of the study, conducted data analysis, and assisted with drafting the manuscript. All authors read and approved the final manuscript.

## Supplementary Material

Additional file 1 Interview Guide. Click here for file
