# Supplementary material for: Strategies for obtaining unpublished drug trial data: a qualitative interview study
Source: Syst Rev. 2013 May 16;2:31. doi: 10.1186/2046-4053-2-31 (PMC3685609; doi:10.1186/2046-4053-2-31)
Supplement: Additional file 1 — Interview Guide. [file 2046-4053-2-31-S1.docx]

**Interview Guide**

Thank you for agreeing to participate in this interview. The purpose of this interview is to understand your experience trying to obtain unpublished data for a meta-analysis/systematic review that you conducted or are conducting. I am going to ask you general questions about obtaining unpublished data as well as questions specific to a certain project. I recognize that you may have tried to obtain unpublished data on multiple occasions for multiple projects, but for the project-based questions please focus on a primary example of a review you conducted where drugs were the intervention.

1. Introduction/Background
2. Briefly describe your academic/research/clinical background as well as the general topics of your reviews.
3. Can you talk about the difference between conducting a Cochrane and a non-Cochrane review?
4. Are drugs generally the intervention in your reviews?
5. Is it standard practice for you to attempt to obtain unpublished data for your reviews?
6. Understanding data - clinical study reports, reviewers’ comments, correspondence and individual patient data

Now I would like to understand your conceptions about data and the world of unpublished data.

1. What is data?
2. What kind of data do you usually work with?
3. Can you discuss or describe the world of unpublished data?
4. How does confidentiality factor in?
5. Can you talk about the difference between unpublished data and unpublished studies as well as the processes trying to obtain them?
6. Why do you feel it is important to use unpublished data?
7. Describe what you believe are the best practices in obtaining unpublished data.
8. Is there a way to make this data more accessible, and whose responsibility do you feel it should be to regulate/manage this access?
9. Obtaining and using the data – Project being discussed

In the following section, I am going to be asking questions about obtaining unpublished data for a particular project. For these questions, please focus on the primary example you chose of a review you conducted where drugs were the intervention. General questions about obtaining unpublished data will follow later in the interview.

1. Describe your experience trying to obtain unpublished data for this review.
2. Why did you decide to search for unpublished data/study?
   - 1. Did you have suspicions the data was available?
     2. Did someone alert you?
3. Where did you look?
   1. Government, Companies, investigators, drug and device regulatory agencies, Manufacturers, Research ethics committees, Institutional review boards, Non-commercial trial register (e.g. clinicaltrials.gov), Company-owned trial register, Trialists/Investigator, Funders, Medical journals, Other How many different feelers did you put out?
4. How long was the process for obtaining the unpublished data?
   1. If long:
      1. Is this generally how long it takes?
      2. Did that impede the completion of the review?
5. How was the quality of the data and was it usable for the review?
6. Can you discuss any confidentiality agreements you subject to and how that affected how you were able to use the data.
7. Can you discuss how the inclusion of the data affected your results/conclusions?
8. What were the main difficulties in incorporating that data into your review?
9. Would you or could you have completed the review without that data?
10. Do you think there was a public health impact due to the delay in obtaining unpublished data, which delayed the completion and publication of the meta-analysis?
11. Was the effort worth the reward?
12. Could you provide me with a citation or reprint of that review?
13. Obtaining the data - General

Now, I would like to ask you some general questions about obtaining unpublished data.

1. Do you have a routine or search strategy that you use when you conduct reviews?
2. Do you have any other agreements/arrangements with other agencies, companies, or researchers within or outside of your country (colleagues in another country who could assist with obtaining data from their own country’s comparable agencies, FDA vs EMA)?
   1. If yes, please describe.
3. If involved in any of these arrangements: Are you subject to any confidentiality agreements that restrict how you can use the data that you receive through one of these arrangements?
4. Can you talk about all of the different types of agencies/organizations/companies where you have obtained unpublished data and how the processes and bureaucracy differ.
5. Have processes changed over time?
6. Can you talk about instances where you were ultimately unable to obtain the unpublished data you wanted?
   1. Was the review completed?
   2. What obstacles did you encounter?
   3. Why do you think you were ultimately unable to obtain the data?
7. Have you tried to obtain unpublished data from a location, and were able to obtain the data for one project but not another?
   1. How did the process at that agency differ from project to project?
8. Have any policy changes affected your ability to obtain unpublished data?
9. What agencies do you feel are the most helpful/forthcoming with their data?
10. Does it get easier to obtain data from an agency where you have previously obtained data?
11. Which agencies are the most difficult to obtain data?
12. Why do you feel these agencies were so reluctant to release the data?
    1. What reasons were you given?
13. How does the process differ between sources of unpublished data?
14. How do you handle requests for information?
15. What advice would you give other researchers searching for unpublished data or just beginning a review?
    1. give guidance for review authors and editorial base staff about how to identify and obtain unpublished data from regulatory agencies, trials registers and web sites.
16. Do you have any other comments you would like to make?
17. Are there any other questions I should be asking?

Thank you very much for participating
